# Interaction of α- and β-zearalenols with β-cyclodextrins

**DOI:** 10.3390/molecules22111910

**Published:** 2017-11-06

**Authors:** Miklós Poór, Afshin Zand, Lajos Szente, Beáta Lemli, Sándor Kunsági-Máté

**Affiliations:** 1 Department of Pharmacology, Faculty of Pharmacy, University of Pécs, Szigeti út 12, H-7624 Pécs, Hungary; af.zand@gmail.com; 2 János Szentágothai Research Center, University of Pécs, Ifjúság útja 20, H-7624 Pécs, Hungary; blemli@gamma.ttk.pte.hu (B.L.); kunsagi@gamma.ttk.pte.hu (S.K.-M.); 3 CycloLab Cyclodextrin Research & Development Laboratory, Ltd., Illatos út 7, H-1097 Budapest, Hungary; szente@cyclolab.hu; 4 Department of General and Physical Chemistry, University of Pécs, Ifjúság útja 6, H-7624 Pécs, Hungary

**Keywords:** zearalenols, mycotoxin metabolites, cyclodextrins, fluorescence spectroscopy, host-guest interactions

## Abstract

Zearalenone (ZEN) is a mycotoxin produced by *Fusarium* fungi. ZEN primarily contaminates different cereals, and exerts a strong xenoestrogenic effect in animals and humans. ZEN is a fluorescent mycotoxin, although molecular interactions and microenvironmental changes significantly modify its spectral properties. During biotransformation, ZEN is converted into α-zearalenol (α-ZOL) and β-zearalenol (β-ZOL), the toxic metabolites of ZEN, which mimick the effect of estrogen. Cyclodextrins (CDs) are host molecules, and have been studied extensively; they can form stable complexes with several mycotoxins, including ZEN. However, information is limited regarding the interactions of CDs with ZOLs. Therefore, we studied the interactions of α- and β-ZOLs with native and six chemically modified β-CDs by fluorescence spectroscopy. Fluorescence enhancement during complex formation, as well as binding constants, were determined. To understand ZOL-CD interactions better, molecular modeling studies were also carried out. Both mycotoxin derivatives formed the most stable complexes with methylated and sulfobutylated CD-derivatives; however, the CD complexes of α-ZOL were significantly stronger than those of β-ZOL. The data presented here indicate which of the chemically modified β-CDs appear more suitable as fluorescence enhancers or as potential mycotoxin binders.

## 1. Introduction

**Figure 1:**
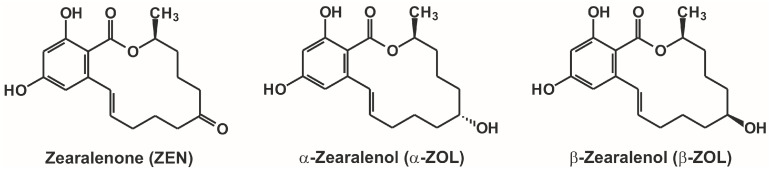
Chemical structures of zearalenone, α-zearalenol, and β-zearalenol.

Zearalenone (ZEN; Figure 1) is a mycotoxin produced by *Fusarium* species [1]. ZEN commonly contaminates a variety of different cereals, including maize, wheat, rye, sorghum, and barley. Furthermore, it also appears in nuts, spices, milk, edible oils, beer, and drinking water [1,2,3]. The wide occurrence of ZEN is associated with its high thermal stability, which makes its elimination from foodstuffs or animal feed difficult [4]. ZEN is a fluorescent mycotoxin. However, changes in the microenvironment (e.g., pH, solvents), as well as its interactions with certain molecules (e.g., cyclodextrins, proteins), can lead to significant spectral changes, which can be examined by fluorescence spectroscopy [5,6]. ZEN expresses a strong estrogenic effect in animals and humans; therefore, it has been classified as an endocrine disruptor molecule [7]. ZEN is rapidly absorbed after oral administration or consumption. Its extensive biotransformation gives rise to its reduced metabolites, α-zearalenol (α-ZOL) and β-zearalenol (β-ZOL; Figure 1), which (along with ZEN) are, in turn, conjugated with glucuronic acid [1,8]. In addition to the liver, some other tissues are also able to form ZOLs from ZEN, including the intestines and even the blood [9,10,11]. Previous studies have reported species-dependent alterations in the hepatic biotransformation of ZEN. For example, ZEN is converted mainly to α-ZOL in pigs; whereas in cattle, β-ZOL is the dominant hepatic metabolite [9,12]. The importance of this alteration is underlined by the finding that α-ZOL is much more toxic than β-ZOL, or even its parent compound ZEN [12,13,14]. In addition, similarly to ZEN, ZOLs can also appear in some foodstuffs or in drinks (e.g., soya meal, milk) as contaminants [15,16].

Cyclodextrins (CDs) have been studied extensively as host molecules in host-guest interactions. The most commonly applied α-, β- and γ-CDs are built up from six, seven, and eight glucose units, respectively. The external surface of cyclodextrins is hydrophilic, whereas their interior is hydrophobic, making it possible to accommodate lipophilic structures or molecules within this internal cavity [17]. Chemically modified CDs are also commonly used, because the stability and selectivity of host-guest complexes are strongly influenced by these modifications [18,19]. Previous studies have reported that several mycotoxins, including aflatoxins, citrinin, ochratoxin A, and ZEN, form stable complexes with native and chemically modified β-CDs [5,20,21,22]. Complex formation of ZEN with β-CDs has been characterized in multiple studies, showing that complex formation leads to substantial increase in the fluorescence signal of ZEN [5,23,24]. It has also been revealed that ZEN-CD interaction can improve the separation and/or quantification of ZEN during its analytical determination [23,25,26,27]. In contrast, the information regarding the interactions of α- and β-ZOLs with CDs is limited. Dall’Asta et al. [23] described the complex formation of ZEN and ZOLs with native β-CD, and reported that the stability of β-ZOL–CD complexes is lower than those of α-ZOL or ZEN. In another study, complex formation of α- and β-ZOLs with β-CD, 2-hydroxypropyl-β-CD, and 2,6-dimethyl-β-CD was investigated, and the dimethylated CD derivative was found to enhance the fluorescence signal of both mycotoxin derivatives most significantly [24]. These findings prompted us to investigate ZOL-CD complex formation further with the involvement of other chemically modified β-CDs. The experiments presented here could aid the development of more appropriate and/or sensitive analytical procedures, and of the means to remove mycotoxin contamination from aqueous solutions suitable for the food industry [28].

In this study, fluorescence spectroscopic investigation of the interactions of α- and β-ZOLs with the native β-cyclodextrin (BCD) and six chemically modified β-CDs was performed, including (2-hydroxypropyl)-β-cyclodextrin (HPBCD), randomly methylated-β-cyclodextrin (RAMEB), heptakis-2,6-di-*O*-methyl-β-cyclodextrin (DIMEB), sulfobutylated β-cyclodextrin (SBBCD), carboxymethyl-β-cyclodextrin (CMBCD), and (2-hydroxy-3-*N*,*N*,*N*-trimethylamino)propyl-β-cyclodextrin (QABCD; or quaternary ammonium β-cyclodextrin). Fluorescence enhancement during the complex formation, as well as the binding constants, was determined. To understand the interactions of ZOL with CDs better, molecular modeling studies were also carried out. The results obtained demonstrate marked differences in the stability of the ZOL-CD complexes studied, and indicate which of the chemically modified β-CD derivatives appears more suitable as fluorescence enhancers or as potential mycotoxin binders.

## 2. Results and Discussion

### 2.1. Effect of pH on the Fluorescence Spectra of α- and β-ZOLs

**Figure 2:**
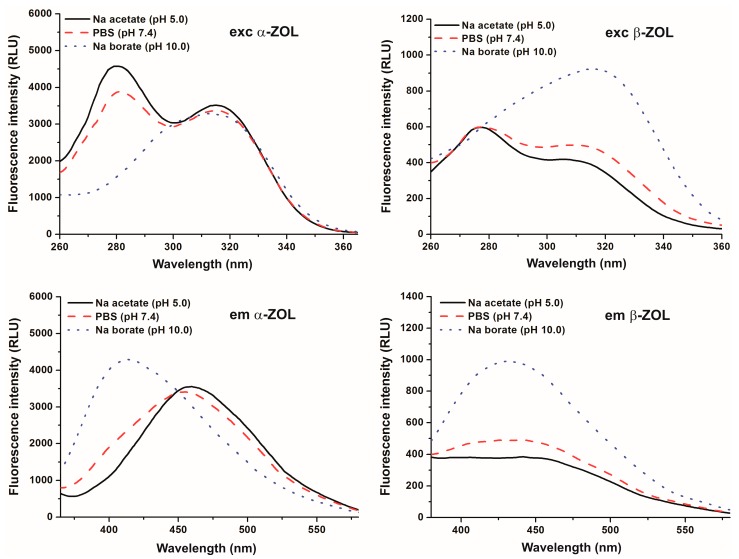
Fluorescence excitation (**top**) and emission (**bottom**) spectra of 20 μM α-ZOL (**left**) and 20 μM β-ZOL (**right**) in 0.05 M sodium acetate (pH 5.0), PBS (pH 7.4), and 0.05 M sodium borate (pH 10.0) buffers (λ_exc_ = 315 nm; λ_em_ = 455 nm).

First, fluorescence excitation and emission spectra of α- and β-ZOLs were examined under different environmental conditions. Similarly to the parent compound [5], the reduced metabolites of ZEN gave two fluorescence excitation peaks at 275 and at 315 nm (Figure 2, top). At acidic and at physiological pH, the 275-nm peak was the dominant with both ZEN derivatives. However, at pH 10.0 the second peak (315 nm) of the excitation spectra became the dominant one, and with β-ZOL a slight redshift could even be observed.

Emission spectra of ZOLs were recorded using 315 nm as the excitation wavelength. At pH 5.0 and 7.4, the emission maximum of both ZOLs was at approximately 455 nm (Figure 2, bottom). The intensity of the emission maximum of β-ZOL was much lower than that of α-ZOL at both pH values. In contrast, the emission signals increased and a marked blueshift was observed in the fluorescence emission wavelength maximum of both α- and β-ZOLs at pH 10.0 (Figure 2, bottom), as the emission maximum was at approximately 410 nm. These data are in good agreement with previous results observed with ZEN [5], and are most likely ascribable to spectral changes resulting from the deprotonation and, therefore, the ionization of ZOLs, similarly to the parent compound.

### 2.2. Effects of CDs on the Fluorescence Spectra of α- and β-ZOLs under Different Circumstances

**Figure 3:**
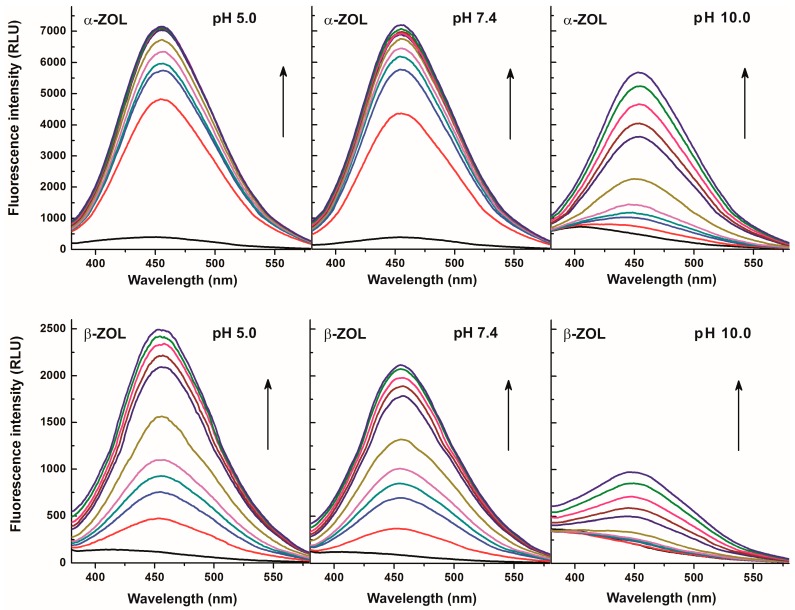
Fluorescence emission spectra of α-ZOL (2 μM; **top**) and β-ZOL (2 μM; **bottom**) in the presence of increasing concentrations of DIMEB (0 mM: black, 0.02 mM: red, 0.05 mM: blue, 0.07 mM: dark cyan, 0.1 mM: magenta, 0.2 mM: dark yellow, 0.5 mM: purple, 0.7 mM: brown, 1.0 mM: pink, 1.5 mM: green, 2 mM: dark blue) in 0.05 M sodium acetate (pH 5.0; left), PBS (pH 7.4; **middle**), and 0.05 M sodium borate (pH 10.0; **right**) buffers (λ_exc_ = 315 nm).

Since the complex formation of ZEN with CDs led to significant enhancement of the fluorescence of the mycotoxin [5], it seemed likely that CDs could increase the fluorescence signals of ZOLs as well. This hypothesis was also supported by some previous studies, where interaction of α- and β-ZOLs with various CDs were investigated [23,24]. In order to characterize ZOL-CD interactions, fluorescence emission spectra of ZOLs were recorded in the absence of CDs and also in their presence at different concentrations ranging from 0.02 to 2.0 mM. Each tested CD increased the fluorescence emission intensities of both α- and β-ZOLs in a concentration-dependent fashion. As Figure 3 demonstrates, fluorescence enhancement of α-ZOL by CDs was similar in sodium acetate (pH 5.0) and in PBS (pH 7.4) buffers with the emission maximum at approximately 455 nm. However, weaker enhancement of the fluorescence signal was observed in borate buffer (pH 10.0), and even the presence of CD concentrations as low as 0.05 mM resulted in a significant redshift of the emission maximum of α-ZOL (410 → 455 nm; Figure 3, top right). The fluorescence spectra of β-ZOL in the presence of CDs were very similar to those observed for α-ZOL (Figure 3, lower panels), although the intensity of the fluorescence was lower. These observations are in good agreement with those obtained with ZEN [5]. The shift of the emission wavelength maximum observed in a buffer of pH 10.0 in the presence of CDs suggests that the ratio of the non-ionized forms of these mycotoxins in the solution increased (Figure 2). These findings point to the fact that CDs can form substantially more stable complexes with non-ionized ZOLs than with the deprotonated mycotoxins, indicating that, despite the alkaline pH, CDs induced a shift in the equilibrium in favor of non-ionized ZOLs during interaction.

Considering the known strong quenching property of water molecules on fluorescence signals of several compounds, the strong enhancement of emissions in our present cases reflects the partial removal of hydration shells of ZOL molecules during their complex formation with CDs [5,21]. The weaker enhancement obtained at higher pH levels and the redshift of the fluorescence spectra in sodium borate buffer (pH 10.0) highlights the fact that CDs interact primarily with the protonated (non-ionized) form of ZOLs. Since the redshift is obtained only at pH 10.0 with α-ZOL, while it was observed even at pH 7.4 with β-ZOL, a much lower *pK_a_* value can be estimated for β-ZOL than for α-ZOL. In order to confirm this hypothesis, *pK_a_* values of ZEN, α-ZOL, and β-ZOL were determined using their excitation spectra recorded at different pH. Based on this evaluation, ZEN and α-ZOL showed the same properties; the *pK_a_* value of both mycotoxins was 7.8, which is close to the previously reported data regarding ZEN [29]. However, a considerably lower acidic dissociation constant of β-ZOL was determined (*pK_a_* = 6.2), suggesting a significantly different extent of ionization of α-ZOL and β-ZOL under physiological as well as under slightly acidic or slightly alkaline circumstances.

### 2.3. Fluorescence Enhancement of ZOLs by Native and Chemically Modified β-CDs

**Figure 4:**
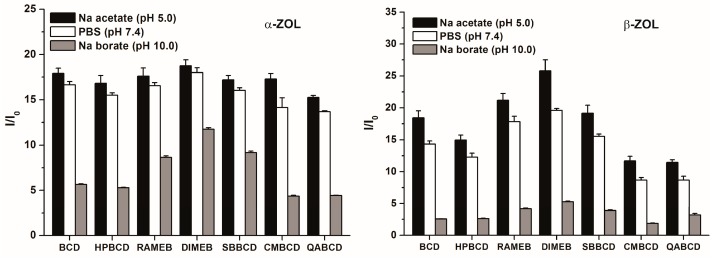
Fluorescence enhancement (*I*/*I*_0_) of α-ZOL (2 μM; **left**) and β-ZOL (2 μM; **right**) in the presence of 2 mM CD concentration in 0.05 M sodium acetate (pH 5.0), PBS (pH 7.4), and 0.05 M sodium borate (pH 10.0) buffers (λ_exc_ = 315 nm, λ_em_ = 455 nm).

The extent of the enhancement of mycotoxin fluorescence brought about by CDs is demonstrated in Figure 4. During these experiments, high CD concentration (2 mM) was used in order to maximize the binding of ZOL molecules to CDs. The chemically modified β-CDs enhanced the fluorescence of α-ZOL similarly to the native BCD (Figure 4, left) with DIMEB appearing slightly stronger, while QABCD appeared slightly weaker compared to the other CDs tested. In contrast, the fluorescence enhancement of β-ZOL by CDs differed substantially, depending on the CD species used, in the following order: DIMEB > RAMEB > SBBCD ~ BCD > HPBCD > CMBCD > QABCD. Irrespective of the CD used, the strongest fluorescence enhancement of both α- and β-ZOLs was observed at pH 5.0, a slightly lower enhancement was found at pH 7.4, and markedly lower fluorescence enhancement was found at pH 10.0. These differences presumably originate from the degree of ionization of these mycotoxins, being partial in PBS (pH 7.4) and practically complete in sodium borate buffer (pH 10.0).

**Figure 5:**
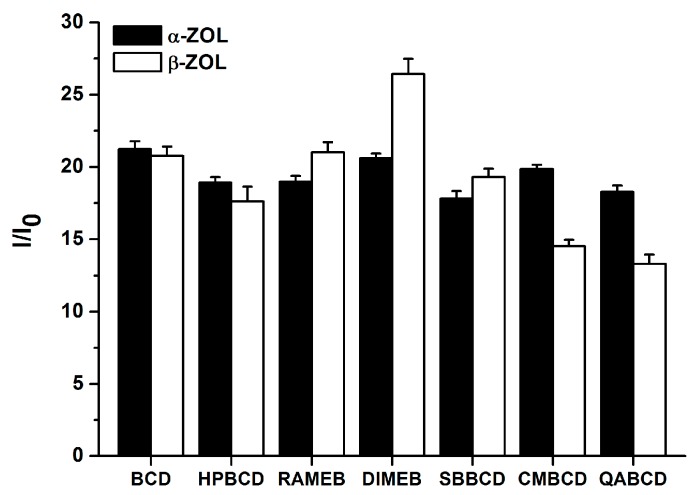
Fluorescence enhancement (I/I_0_) of α-ZOL (2 μM) and β-ZOL (2 μM) in the presence of 2 mM CD concentration in 0.05 M sodium acetate (pH 5.0) buffer (λ_exc_ = 275 nm, λ_em_ = 455 nm).

In the next experiments, fluorescence enhancement of ZOLs by CDs was investigated in sodium acetate buffer (pH 5.0) using an excitation wavelength of 275 nm. This excitation wavelength results in stronger emission signals (compared to excitation at 315 nm), and thus it is preferred for the analytical utilization of ZOL-CD complex formation. As presented in Figure 5, the relative enhancements of the fluorescence signals of ZOLs are very similar to those observed in the previous experiments carried out at 315 nm excitation wavelength, and only minor differences were found.

### 2.4. Binding Constants of ZOL-CD Complexes

**Figure 6:**
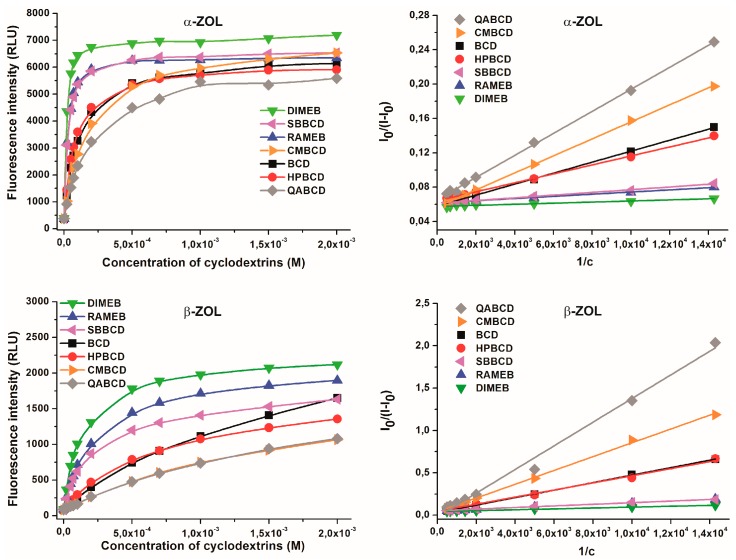
Fluorescence emission intensities of α-ZOL (2 μM; **top left**) and β-ZOL (2 μM; **bottom left**) in the presence of increasing CD concentrations (0–2 mM), and Benesi-Hildebrand plots of α-ZOL–CD (**top right**) and β-ZOL–CD (**bottom right**) complexes in 0.05 M sodium acetate buffer (pH 5.0; λ_exc_ = 315 nm, λ_em_ = 455 nm).

Binding constants of ZOL-CD complexes were determined in sodium acetate buffer (pH 5.0) at 315 nm excitation wavelength. As Figure 6 demonstrates, the presence of increasing CD concentrations resulted in the gradual increase in the fluorescence signals. Based on the intensity of fluorescence emission of ZOLs (both at 2 μM) measured at 455 nm in the presence of increasing CD concentrations (0–2 mM), binding constants of ZOL-CD complexes were calculated by employing the graphical application of the Benesi-Hildebrand equation (Equation (1); given in the Materials and Methods section). Benesi-Hildebrand plots exhibited excellent fitting with the *n* = 1 model, suggesting the 1:1 stoichiometry of ZOL-CD complex formation (Figure 6). The calculated log*K* values of ZOL-CD complexes (as well as the data reported previously for ZEN-CD interactions [5]) are listed in Table 1. Interestingly, the stability constants of α-ZOL–CD complexes were comparable with those of ZEN-CD interactions reported previously [5]; whereas for β-ZOL, the binding constants appeared significantly lower. Each of the examined CD complexes of β-ZOL exhibited a lower binding constant by approximately one order of magnitude compared to α-ZOL or to the parent compound ZEN. Furthermore, both α- and β-ZOLs formed the most stable complex with DIMEB, followed by RAMEB and SBBCD with slightly lower binding constants. Markedly lower stability values arose for ZOL-CD complexes in the presence of HPBCD and BCD, while the lowest binding constants were observed with CMBCD and QABCD. These results indicate that, despite the remarkable differences between the binding constants of α-ZOL–CD and β-ZOL–CD complexes, the same chemical modification of CDs provides these molecules with the same tendency to form complexes with ZOLs (and ZEN) with respect to their stability constants.

In order to confirm our results calculated using the Benesi-Hildebrand plot, binding constants were also determined by non-linear fitting employing the Hyperquad2006 program (see details in Section 3.2). As Table 2 demonstrates, slightly higher but similar log*K* values were calculated with Hyperquad program than with the Benesi-Hildebrand plot. Furthermore, this evaluation also suggests 1:1 stoichiometry of complex formation and both evaluations show the same tendencies regarding the comparison of the stabilities of ZOL-CD complexes (Table 1 and Table 2).

The data presented above clearly indicates that α-ZOL forms highly stable complexes with β-CDs, similarly to its parent compound ZEN. The binding constants of β-ZOL–CD complexes are significantly lower; nevertheless, these interactions are still strong and have decent stability, especially for complexes of methylated and sulfobutylated CD derivatives. Based on these data, we can emphasize that methylated and sulfobutylated β-CDs seem the most promising binders of ZOLs and ZEN in vitro. This may be of great importance, because Appell and Jackson [28] have proposed the applicability of insoluble CD-polymers as a means of decreasing the toxin content of mycotoxin-contaminated drinks. In their study, the ochratoxin A concentrations of aqueous solutions and wine were significantly reduced by a β-CD-polyurethane polymer. Moreover, it is also noteworthy that the binding constants of α-ZOL–BCD and ZEN–BCD complexes are more than 50-fold higher, compared to the binding constant of ochratoxin A–BCD complex [5,22], arguing for the suitability of the studied interactions in removing mycotoxin contamination from food and drinks. 

As described previously, BCD reduced the toxicity of ZEN on liver cells [5], suggesting that CDs may be suitable even for detoxification, owing to the inhibition of cellular toxin uptake. However, the cellular toxicity of some CDs may also be of concern. Methylated CD derivatives have relatively high cytotoxicity compared to other CDs, which is presumably due to their very strong interaction with cholesterol [30,31]. In this study, we demonstrated that SBBCD forms similarly stable complexes with ZOLs to methylated CDs (DIMEB or RAMEB). As sulfobutylated β-CDs are significantly less toxic than the methylated derivatives [30], it is reasonable to hypothesize that these derivatives appear more promising as a means of in vivo detoxification. However, further in vitro and in vivo studies are necessary to confirm or reject this hypothesis.

### 2.5. Molecular Modeling Studies

It is well known that, during the formation of inclusion complexes of small molecules with β-CDs, guest molecules partly lose their solvation shells prior to or during the inclusion. The whole process is significantly influenced by this phenomenon. To obtain an appropriate model, two series of calculations were performed: in the first, dehydration of the guest molecules calculated using the TIP3P model was considered (Table 3); in the second, the effect of dehydration was not considered during the calculation of enthalpy and entropy changes (Table 4). The few tens negative Gibbs free energy changes confirm the formation of stable ZOL-CD complexes at room temperature. Similar Gibbs free energy changes are associated with the molecular interactions of ZOLs with CDs, irrespective of considering the solvation enthalpy and entropy of ZOLs. When comparing Gibbs free energy changes associated with the complex formation of CDs with ZOL, the molecules give similar values irrespective of the guest molecules losing or retaining their solvation shells prior to the complex formation. The enthalpy and entropy terms, however, reveal significant differences in thermodynamic properties. If ZOL molecules lose their solvation shells before the complex formation (Table 3), then the interaction is an entropy-driven process. In contrast, the complexation becomes an enthalpy-driven process if we hypothesize that ZOL molecules enter into the cavity of CDs with their solvation shells. This feature reflects the presence of enthalpy-entropy compensation, according to the solvation shell of ZOLs. However, the van’t Hoff equation highlights that the temperature dependence of the stability constants is determined by the enthalpy term of the interaction. As a result, the Gibbs free energy shows stronger temperature dependence when the dehydration of ZOL molecules does not occur prior to the entry of the mycotoxin into the cavity of the CD.

To obtain the potential background of different affinities of ZOL-CD complexes, electron distribution of CD cavities regarding native and substituted CDs was calculated at AM1 level. Results suggest less than one percent difference in charge densities, which confirms a negligible role of the electronic distribution of the CD skeleton. This is not surprising, if we consider the sigma-bonded skeleton of CD molecules. Furthermore, the substituents are applied in the outer region of the CD skeleton; accordingly, steric effects can also be neglected. However, it is conspicuous in Figure 4 that stability of CD complexes of both α- and β-ZOLs showed the same tendencies for the sizes and average numbers of substituents used. Our previous observations on a similar system showed decreasing entropy when a host molecule with a more flexible skeleton takes part in the molecular association [32]. The interaction of ZOL molecules with CDs reduces the flexibility of the molecular skeleton of CDs, and the reduced motion decreases the vibrational entropy, which is important regarding the Gibbs free energy term.

The static QC calculations at AM1 level confirm the interaction of the aromatic OH group to one oxygen atom located in the lower rim of the molecular skeleton. This bond is slightly weaker with β-ZOL than with α-ZOL. This property itself could support the experimental data; however, our molecular dynamics simulations highlight the potential importance of temperature-dependent molecular motions during the complex formation. Complex stabilities are affected either by the binding between the molecules interacted (enthalpy term) and the changes of molecular motions, which are preferably affected by the changes of molecular vibrations (entropy term). Since these two effects compete with each other (the variation of ΔH term regarding the different substitution of CDs are in the same range as the variation of the ΔS term around room-temperature; see Table 4), we are unable to find a general correlation between the molecular structure and the stabilities. 

**Figure 7:**
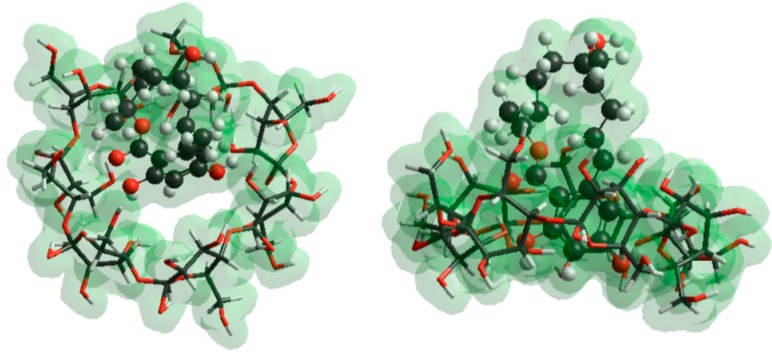
Top and side views of BCD–α-ZOL complex. Aromatic moiety of ZOL molecules is preferred for inclusion interaction with BCD cavity.

The comparison of the experimentally determined binding constants of α-ZOL–CD and β-ZOL–CD complexes suggests the significantly higher stability of CDs with α-ZOL molecules than with β-ZOL (Figure 7). Both ZOL enantiomers yielded the following order of complex stability constants: DIMEB > SBBCD > BCD. These observations are consistent with the results of theoretical calculations describing the formation of α-ZOL–CD complexes, also yielding the stability order DIMEB > SBBCD > BCD. Furthermore, theoretical calculations support the experimental observation that the DIMEB molecules form considerably stronger complexes with the α-ZOL enantiomer. However, theoretical results suggest racemic composition of α-ZOL–CD and β-ZOL–CD complexes at room-temperature, while formation of slightly stronger complexes is observed in the case of the β-ZOL–SBBCD complex. To get a plausible description for this observation, transition states with the associated activation energies were calculated in the six possible cases (formation of complexes of both ZOL enantiomers with the three CD derivatives). The results show that the formation of β-ZOL–SBBCD complex requires much higher activation energy than the formation of the five other complexes (Table 5). Considering the activation energy of β-ZOL → α-ZOL transition, it is a plausible explanation that a β-ZOL → α-ZOL transition occurs during the entry of β-ZOL molecules into the cavity of SBBCD. Although it highlights the role of kinetic processes during complex formation, spectroscopic data do not support this theory, because the β-ZOL–SBBCD complex shows much lower fluorescence intensities compared to the fluorescence signal of α-ZOL–SBBCD complex.

## 3. Materials and Methods

### 3.1. Reagents

The mycotoxins α-zearalenol (α-ZOL) and β-zearalenol (β-ZOL) were purchased from Sigma-Aldrich. Native and chemically modified cyclodextrins, including β-cyclodextrin (BCD), (2-hydroxypropyl)-β-cyclodextrin (HPBCD), randomly methylated-β-cyclodextrin (RAMEB), heptakis-2,6-di-*O*-methyl-β-cyclodextrin (DIMEB), sulfobutylated β-cyclodextrin (SBBCD), carboxymethyl-β-cyclodextrin (CMBCD), and (2-hydroxy-3-*N*,*N*,*N*-trimethylamino)propyl-β-cyclodextrin (QABCD; or quaternary ammonium β-cyclodextrin), were obtained from Cyclodextrin Research & Development Laboratory Ltd. Stock solutions of ZOLs (5000 μM) were prepared in ethanol (Reanal, spectroscopic grade) and stored at −20 °C protected from light.

### 3.2. Fluorescence Spectroscopic Measurements

Steady-state fluorescence measurements were performed employing a Hitachi F-4500 fluorescence spectrophotometer. Interactions of α- and β-ZOLs with CDs were examined in 0.05 M sodium acetate (pH 5.0), in PBS (pH 7.4), and in 0.05 M sodium borate (pH 10.0) buffers at 25 °C, in the presence of air. When testing the effects of CD concentration on the complex formation followed by the enhancement of fluorescence, CDs were applied at concentrations 0, 0.02, 0.05, 0.07, 0.10, 0.20, 0.50, 0.70, 1.0, 1.5 and 2.0 mM together with 2 μM α-ZOL or 2 μM β-ZOL. The fluorescence emission spectra were recorded using the excitation wavelengths of 275 nm or 315 nm (λ_em_ = 455 nm). Binding constants (*K*, with the dimension of dm^3^/mol) of ZOL-CD complexes were calculated employing the graphical application of the Benesi-Hildebrand equation assuming 1:1 stoichiometry:

(1) $$\frac{I_{0}}{\left(I-I_{0}\right)}=\frac{1}{A}+\frac{1}{A\;\times \;K\;\times \;{\left[CD\right]}^{n}}$$

where *I_0_* and *I* denote the fluorescence emission intensities of ZOLs in the absence and in the presence of CDs, respectively; [*CD*] is the molar concentration of the host molecule, while *A* is a constant and *n* is the number of binding sites.

To confirm the results determined by the Benesi-Hildebrand plot, *K* values were also evaluated with the Hyperquad2006 program package. Binding constants were calculated by non-linear fitting, based on the fluorescence emission data obtained for experiments performed at pH 5.0 (sodium acetate buffer). To calculate *K* values associated with the ZOL-CD complex formation, the following equations are implemented in the Hyperquad code:

(2) $$pCD+qZOL↔CD_{p}ZOL_{q}$$

(3) $$K_{pq}=\frac{\left[CD_{p}ZOL_{q}\right]}{{\left[CD\right]}^{p}{\left[ZOL\right]}^{q}}$$

where *p* and *q* are the coefficients that indicate the stoichiometry associated with the possible equilibria in the solution. In Hyperquad2006 computer fitting program all equilibrium constants are defined as overall association constants:

(4) $$CD+ZOL↔CDZOL\;\beta_{1}=\frac{\left[CDZOL\right]}{\left[CD\right]\left[ZOL\right]}\;$$

(5) $$CD+2ZOL↔CDZOL_{2}\;\beta_{2}=\frac{\left[CDZOL_{2}\right]}{\left[CD\right]{\left[ZOL\right]}^{2}}$$

(6) $$CD+qZOL↔CDZOL_{q}\;\beta_{q}=\frac{\left[CDZOL\right]}{\left[CD\right]{\left[ZOL\right]}^{q}}$$

The relationship between the overall association constants and the stepwise association constants calculated by Hyperquad is the following:

(7) $$\beta_{1}=K_{1};\;\beta_{2}=K_{1}\times K_{2};\;\beta_{q}=K_{1}\times K_{2}\ldots \times K_{q}$$

The stoichiometry and association constants of the complexes were determined by the model associated with the lowest standard deviation.

The *pK_a_* values of α-ZOL, β-ZOL, and ZEN were determined by measuring the molar coefficients, reflecting the concentration dependence of the intensities of excitation and the excitation spectra associated with the pH value close to the estimated *pK_a_* (λ_em_ = 455 nm).

### 3.3. Molecular Modeling Studies

In the molecule of DIMEB, two methyl groups were added to replace the hydrogens of the OH groups at the positions 2 and 6 in each glucose unit. In SBBCD, the sulfobutyl groups were added randomly during the synthesis; therefore, all OH groups were considered equivalent while preparing the model structure. Thus, at the lower rim four sulfobutyl groups replaced the hydrogen atom of the OH group at position 6. At the upper rim, OH groups at positions 2 and/or 3 were replaced by *O*-sulfobutyl moieties, eight substituents altogether.

According to previous studies [21,32], the initial structures of the interacting species were determined at semi-empirical level using AM1 approximation. Then, atomic charges of the two enantiomers of zearalenol (α-ZOL and β-ZOL) and the CDs were calculated using the same B3LYP/6-31G(d) method, and the basis was set by performing natural population analysis (NPA).

Transition states during the reaction were determined by the HyperChem package, and existence of the saddle point was validated by the appearance of one virtual vibration frequency.

Molecular dynamics simulations with (TIP3P model) and without explicit water molecules were used to determine the initial structures of the three CDs (BCD, DIMEB, and SBBCD). MM + force field implemented in the HyperChem 8.0 program was applied for these calculations. 

The entropy term of molecular interactions was considered as a change of the molecular vibrations [33]. The overall effect of the vibrations on the entropy changes and the vibrational entropy content of each species were calculated by applying the Boltzmann statistics. Accordingly, the frequencies were calculated by harmonic approximation, and the entropy was calculated in the standard way using HyperChem code:

(8) $$S_{vib}=R\sum_{i}\{\frac{h\nu_{i}/kT}{e^{\left(h\nu_{i}/kT\right)}-1}-\ln[1-e^{\left(-h\nu_{i}/kT\right)}]\}$$

where *ν_i_* is the frequency of vibration, *T* is the temperature (here equal to 298.16 K). Considering the error, which may be caused by extrapolations [34], the results were compared within the temperature range of experiments only.

The solvation entropy and enthalpy of ZOL enantiomers were calculated at a semiempirical AM1 level using the TIP3P model, implemented in the HyperChem code; entropy content calculated for the gas phase molecule was subtracted from the entropy term of solvated species calculated by the TIP3P model. A similar method applied for the enthalpy term. 

## 4. Conclusions

In this study, the interactions of α- and β-ZOLs with β-CDs were examined by fluorescence spectroscopy and molecular modeling studies. ZOLs formed stable complexes with each tested CD. However, the stability of the different ZOL-CD complexes altered markedly. Both α- and β-ZOLs formed the most stable complexes with methylated (DIMEB and RAMEB) and sulfobutylated (SBBCD) CD-derivatives. Although the stability of ZEN-CD and α-ZOL–CD complexes was comparable, α-ZOL formed significantly more stable complexes with CDs compared to β-ZOL. Based on these results, β-CDs are strong enhancers of the fluorescence of ZOLs, indicating the formation of ZOL-CD complexes. The stability of ZOL-CD complexes seems promising in the development of new decontamination and/or detoxification methods, employing CDs as binders of these mycotoxins.

## Figures and Tables

**Table 1:** Decimal logarithmic values of binding constants (*K*; dm^3^/mol) of ZOL-CD complexes determined using the graphical application of Benesi-Hildebrand equation (Equation (1)) in 0.05 M sodium acetate buffer (pH 5.0; λ_exc_ = 315 nm, λ_em_ = 455 nm).

	BCD	HPBCD	RAMEB	DIMEB	SBBCD	CMBCD	QABCD
**ZEN** ^1^	3.99 ^1^	3.94 ^1^	4.36 ^1^	4.76 ^1^	4.20 ^1^	- ^2^	3.75 ^1^
**α-ZOL**	3.90 ± 0.01	4.05 ± 0.02	4.59 ± 0.04	4.89 ± 0.02	4.58 ± 0.00	3.69 ± 0.02	3.65 ± 0.04
**β-ZOL**	2.79 ± 0.02	3.11 ± 0.04	3.68 ± 0.01	3.88 ± 0.00	3.64 ± 0.02	2.65 ± 0.05	2.25 ± 0.09

^1^ based on Poór et al. [5]; ^2^ no data available.

**Table 2:** Decimal logarithmic values of binding constants (*K*; dm^3^/mol) of ZOL-CD complexes determined by non-linear fitting with the Hyperquad program (Equations (2)–(7)) in 0.05 M sodium acetate buffer (pH 5.0; λ_exc_ = 315 nm, λ_em_ = 455 nm).

	BCD	HPBCD	RAMEB	DIMEB	SBBCD	CMBCD	QABCD
**α-ZOL**	4.03 ± 0.04	4.21 ± 0.01	4.74 ± 0.01	5.00 ± 0.01	4.68 ± 0.01	3.92 ± 0.02	3.88 ± 0.02
**β-ZOL**	3.08 ± 0.01	3.41 ± 0.02	3.82 ± 0.02	4.02 ± 0.02	3.85 ± 0.02	3.07 ± 0.01	2.64 ± 0.01

**Table 3:** Thermodynamic parameters of ZOL-CD interactions considering the dehydration of the guest molecules prior to the complex formation (based on modeling studies).

**α-ZOL**	**ΔH (kJ/mol)**	**ΔS (J/K·mol)**	**ΔG_298K_ (kJ/mol)**
BCD	−23.4	34.6	−33.8
DIMEB	−32.4	24.3	−39.7
SBBCD	−25.4	32.5	−35.1
**β-ZOL**	**ΔH (kJ/mol)**	**ΔS (J/K·mol)**	**ΔG_298K_ (kJ/mol)**
BCD	−23.4	34.6	−33.8
DIMEB	−19.6	48.5	−34.1
SBBCD	−27.4	31.4	−36.8

**Table 4:** Thermodynamic parameters of ZOL-CD interactions without considering the dehydration of the guest molecules prior to the interaction (based on modeling studies).

**α-ZOL**	**ΔH (kJ/mol)**	**ΔS (J/K·mol)**	**ΔG_298K_ (kJ/mol)**
BCD	−37.6	−7.0	−35.6
DIMEB	−46.6	−17.3	−41.5
SBBCD	−39.6	−9.1	−36.9
**β-ZOL**	**ΔH (kJ/mol)**	**ΔS (J/K·mol)**	**ΔG_298K_ (kJ/mol)**
BCD	−37.6	−7.0	−35.6
DIMEB	−33.8	6.9	−35.9
SBBCD	−41.6	−10.2	−38.6

**Table 5:** Activation energies associated with the formation of ZOL-CD complexes (based on modeling studies).

α-ZOL	E_a_ (kJ/mol)	β-ZOL	E_a_ (kJ/mol)
BCD	−11.6	BCD	−11.9
DIMEB	−13.7	DIMEB	−13.4
SBBCD	−12.2	SBBCD	−22.2
